# Identification Tests of Modern Vehicles’ Electromagnetic Environment as Part of the Assessment of Their Functional Safety

**DOI:** 10.3390/s25010007

**Published:** 2024-12-24

**Authors:** Daniel Sterniczuk, Weronika Zaklika, Maciej Kozłowski

**Affiliations:** 1 Łukasiewicz Research Network—Automotive Industry Institute (Łukasiewicz—PIMOT), Jagiellońska 55 Street, 03-301 Warsaw, Poland; daniel.sterniczuk@pimot.lukasiewicz.gov.pl; 2 Faculty of Transport, Warsaw University of Technology, 00-665 Warsaw, Poland; maciej.kozlowski@pw.edu.pl

**Keywords:** electromagnetic compatibility, automotive safety, functional safety, EMC, electromagnetic environment

## Abstract

Are the regulations relating to electromagnetic compatibility (EMC) sufficient to ensure the safety of all autonomy systems? EMC is one of the critical factors influencing the proper functioning of a vehicle and its safety. However, the safety of autonomous vehicles from the perspective of EMC has not been comprehensively researched to date. The purpose of this article is to evaluate whether the currently imposed requirements are adequate. To assess research methods and requirements, it is necessary to determine the electromagnetic environment of vehicles. For this purpose, identification tests of the vehicle’s operating environment were conducted. The article presents the results of research on both the internal and external electromagnetic environments of vehicles. These studies were carried out using normative methods. Despite the significant importance of EMC and the existence of defined normative requirements, the results of the conducted research indicate that some devices available on the market, including those unrelated to vehicle autonomy, fail to meet these standards. Consequently, devices responsible for vehicle safety must be resistant to such electromagnetic exposures. In extreme cases, the malfunction of a single vehicle component due to inadequate electromagnetic compatibility could lead to the disruption of vehicle functions or driver disorientation. Such occurrences pose a direct threat to road traffic safety and the well-being of passengers.

## 1. Introduction

**Figure 1:**
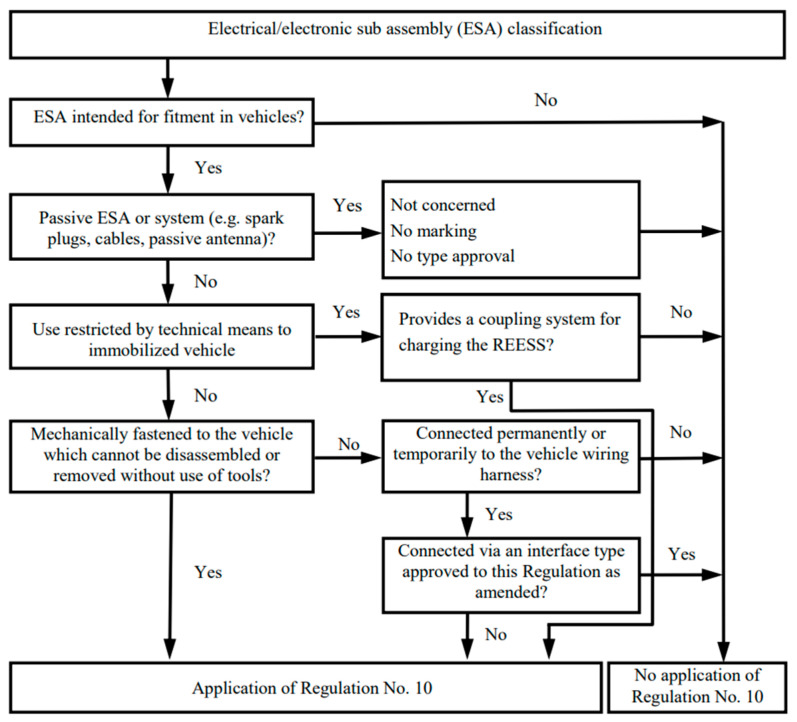
Scheme of application of UN Regulation No. 10 [5].

The dynamic evolution of the automotive industry—from combustion vehicles, through hybrid and electric vehicles, to automated vehicles—has introduced new research challenges [1,2]. Autonomous vehicles have become an increasingly prominent topic within the automotive sector [2], as they are widely regarded as the future of transportation. However, to enable their implementation and widespread adoption, comprehensive testing is required to confirm their proper operation and safety. Currently, the market predominantly focuses on conducting road tests for autonomous and automated vehicles. While road tests are essential, they are not sufficient. Many additional factors must be examined to ensure the safety of autonomous vehicles. One critical issue is electromagnetic compatibility (EMC) [3]. Current vehicle designs sometimes lead to EMC problems. The integration of autonomous systems, combined with their interaction with electric vehicle installations, adds a level of complexity that raises concerns about whether current requirements and assessment methods sufficiently evaluate such complex systems [4]. Additionally, it is worth noting that autonomous systems are often even more critical to vehicle safety. The current state of knowledge regarding safety requirements for vehicles and their equipment is defined in the UN Regulations. Compliance with these regulations is mandatory for introducing a vehicle or its components to the market. The regulation addressing electromagnetic compatibility (EMC) is UN Regulation No. 10; however, it does not specifically address the unique issues associated with autonomous vehicles. This regulation defines devices subject to EMC testing as all electrical and electronic devices installed in vehicles and connected to the vehicle’s electrical system. A diagram illustrating the application of this regulation to individual objects is presented in Figure 1.

From the above diagram, it is evident that all autonomy systems used in vehicles must comply with the requirements of these regulations. However, are these requirements sufficient to ensure the safety of all autonomy systems? Electromagnetic compatibility (EMC) is one of the critical elements influencing the proper functioning of vehicles and, consequently, their safety. This analysis extends to conventional cars as well, given that all modern vehicles, including combustion and hybrid cars, are now equipped with advanced driver assistance systems (ADAS). For many years, vehicle manufacturers have directed their efforts toward developing electronic systems, including hundreds of sensors and actuators designed to protect and assist the driver during operation. These systems also function as entertainment components (e.g., audio and video systems). In the latest designs, electrical and electronic devices are used to control essential vehicle functions, such as steering and emergency braking [6,7,8,9,10,11]. Malfunctioning of these systems can disorient the driver and, in extreme cases, eliminate the driver’s ability to control the vehicle [12]. Such situations can result in accidents, posing direct threats to the health and lives of drivers and passengers [13,14].

Moreover, there is also a consensus that the vehicle of the future will be electric, although there is no agreement on the type of primary energy source. The current trend leans toward hydrogen; however, most systems in modern vehicles are electric.

**Figure 2:**
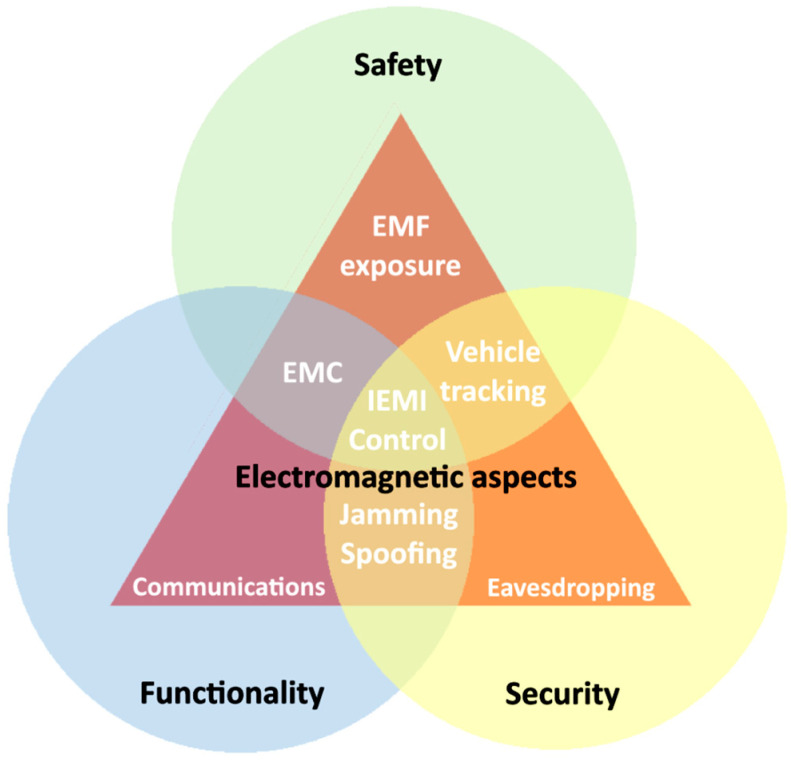
The importance of electromagnetic aspects for vehicle safety, security and functionality [6].

To minimize the risk of errors in the functioning of electrical and electronic devices, component manufacturers often consider EMC issues during the design phase. Pre-qualification tests are then conducted to refine prototypes and ensure reliable operation in specific electromagnetic environments [15,16]. This proactive approach enhances vehicle safety. Unfortunately, due to errors in interpreting regulations, some peripheral devices enter the market without sufficient understanding of their electromagnetic compatibility or their impact on critical vehicle systems. An increasing focus is now being placed on a holistic design approach. Risk analyses are becoming integral to design processes, with particular emphasis on functional safety. However, the automotive industry’s adoption of functional safety practices from other sectors has been limited. This limitation stems from the widespread belief that meeting basic EMC requirements is sufficient to eliminate safety problems related to electromagnetic compatibility—a belief that is, unfortunately, incorrect. The significance of EMC for vehicle safety is illustrated in Figure 2. The complexity of modern vehicles is growing faster than the ability to manage them with current methods and tools. Many manufacturers face challenges in integrating individual electronic components into cohesive systems, and some of these challenges have even led to vehicle recalls [13]. EMC-related issues are increasingly driving manufacturers to rethink system designs and focus on enhancing electromagnetic immunity and functional safety. In response to these challenges, IEEE developed a document outlining the correct process for designing electronic devices with respect to EMC and functional safety [17]. However, while this change in design philosophy is a step forward, it does not address the lack of verification requirements. Manufacturers alone cannot bear the full responsibility for ensuring the safety of autonomous vehicles and their systems. New research methods, requirements, and evaluation criteria must be developed to enable manufacturers to verify the operation, durability, and resistance of their products. At the same time, consumers have the right to purchase fully tested and verified products, assessed by external, independent entities rather than solely by the manufacturer.

There is currently a well-established view that the car of the future will be electric.

## 2. Materials and Methods

**Figure 3:**
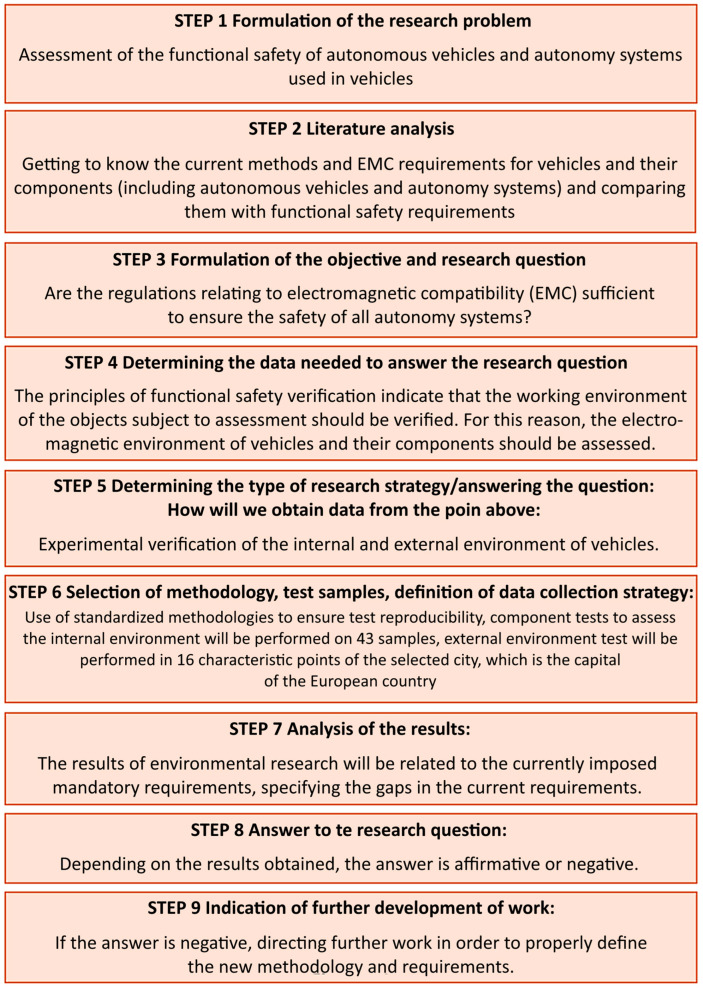
Research design.

The research design is presented in Figure 3.

### 2.1. Functional Safety and Electromagnetic Compatibility

The safety of autonomous vehicles and their detection systems from the perspective of electromagnetic compatibility (EMC) has not yet been comprehensively researched. The first step towards developing new research methods and requirements is to determine the electromagnetic environment of vehicles. Identifying the parameters of the operating environment is a critical element necessary for the subsequent verification of correct vehicle operation [18]. For most electrical and electronic devices, defining all elements that influence their performance in the operating environment is a significant challenge.

The correct process of defining the operating environment involves the following steps:

- creating a list of initial questions regarding the location of the equipment;
- considering future technological trends;
- evaluating future changes in the environment;
- assessing foreseeable electromagnetic hazards due to electrical faults, misuse, and intentional electromagnetic interference (IEMI);
- comparing predictable electromagnetic threats with equipment technologies to identify where EM environment investigation is particularly required (based on the criticality of the safety application);
- conducting an in-depth examination of environmental aspects;
- writing a quantitative engineering specification for the EM environment throughout the product lifecycle [19].

When performing this analysis, the focus should be on the worst-case scenario that could occur. Developing such a verification process is already complex for stationary devices with specific operating environments. However, vehicles are especially vulnerable to electromagnetic disturbances due to the dense placement of electronics in close proximity to each other and a single power source. Additionally, the vehicle’s electromagnetic environment is difficult to verify, as it is a variable factor influenced by vehicle movement.

Thus, the problem of verifying the vehicle operating environment can be divided into two stages. The first stage involves selecting characteristic locations that may represent larger areas, assuming higher field strengths in these locations. Literature analysis suggests that places potentially characterized by higher field intensity include, but are not limited to, the following [20,21,22]:

- railway stations,
- digital terrestrial television antennas,
- wi-fi transmission antennas,
- radio antennas,
- high-voltage transmission lines,
- power transformers,
- mobile phone base stations,
- high-density urban areas or service zones.

Another important issue involves threats in the operating environment of autonomous systems that are not directly related to EMC problems. For instance, high temperatures pose a significant challenge. Normative documents specify EMC tests in a constant climatic environment, but the behavior of an object exposed to both high temperature and electromagnetic fields simultaneously is not tested [17]. Similarly, exposure to vibrations during electromagnetic testing is not addressed. Mandatory tests do not provide any insight into how the object will behave when subjected to vibrations and electromagnetic waves of specific intensity simultaneously [23]. Additional parameters that should be considered include:

- resistance to solar radiation,
- resistance to bending and crushing,
- resistance to humidity and condensation,
- resistance to pollution,
- wear resistance,
- resistance to cyclical exposures occurring separately or concurrently.

Another unverified aspect is the impact of prolonged use of electrical devices on changes in their electromagnetic compatibility. Currently, mandatory EMC tests in the automotive industry are conducted only once, prior to market introduction. Unfortunately, there are no requirements for inspecting such devices after a specified period. Furthermore, tests simulating the aging process of the object are not planned. Since electronic components are degradable, it is reasonable to suspect a decline in their efficiency over time [23].

### 2.2. Identification Tests of the Electromagnetic Environment of Vehicles and Their Systems

The electromagnetic environment associated with a vehicle is influenced by multiple factors, including the electromagnetic field surrounding the vehicle, which is emitted by infrastructure facilities and other vehicles; the electromagnetic field inside the vehicle, which is the sum of emissions from the vehicle’s systems and installations; and any transient states occurring within the vehicle’s electrical installation. Defining a representative electromagnetic environment requires identification tests of the EM field in the operational environment of the vehicle, as well as tests of individual components. It also involves estimating the frequency range and amplitude of disturbances, which represent the combined emissions of electrical and electronic components installed in the vehicle.

For the purposes of this study, identification tests were conducted to analyze the electromagnetic environment in the city of Warsaw, supplemented by test results of electrical and electronic systems currently installed in vehicles. Measurements were conducted under standardized conditions to ensure repeatability. This approach was also employed to minimize the likelihood of errors in statistical evaluation. The nature of the EMC phenomenon, the applied measurement equipment, and the use of normative methods ensured the exclusion of gross errors during measurements.

#### 2.2.1. Internal Electromagnetic Environment of Vehicles Based on Tests of Electrical/Electronic Systems

**Figure 4:**
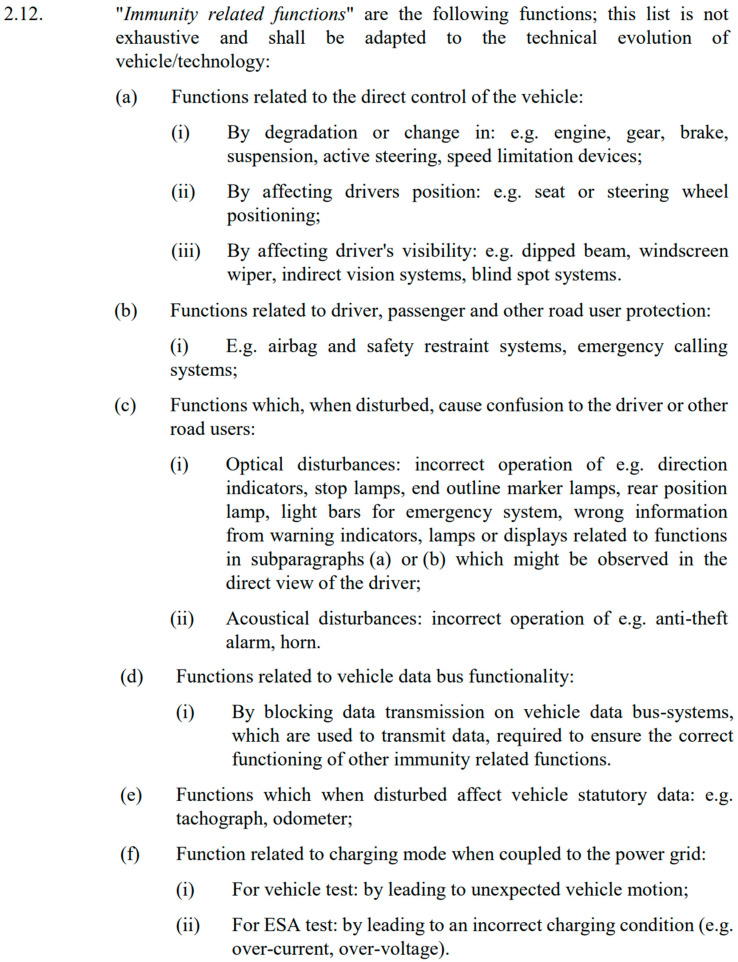
Section 2.12. UN Regulation No. 10.06 [5].

A selection of devices that may impact functions related to immunity was made for testing. Improper installation in the vehicle or a lack of electromagnetic compatibility could compromise the safety of the vehicle. The so-called immunity-related functions are defined in UN Regulation No. 10.06. This definition is presented in Figure 4, which contains a scan of an excerpt from the cited document.

The parameters of the method for measuring radiated broadband and narrowband electromagnetic emissions from electrical and electronic components, in accordance with the requirements of UNECE Regulation No. 10 and the CISPR 25 standard [24], are presented in Table 1, Table 2, Table 3 and Table 4. The measurement uncertainty in the frequency range 30–200 MHz was estimated at 5.45 dB and in the frequency range 200–1000 MHz at 5.98 dB.

**Figure 5:**
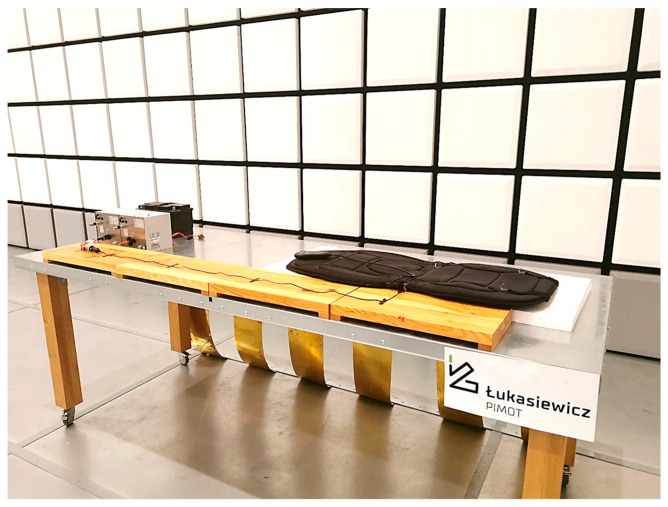
Photo of the test bench for measuring radiated electromagnetic emissions (example of DUT: massage seat cover).

**Figure 6:**
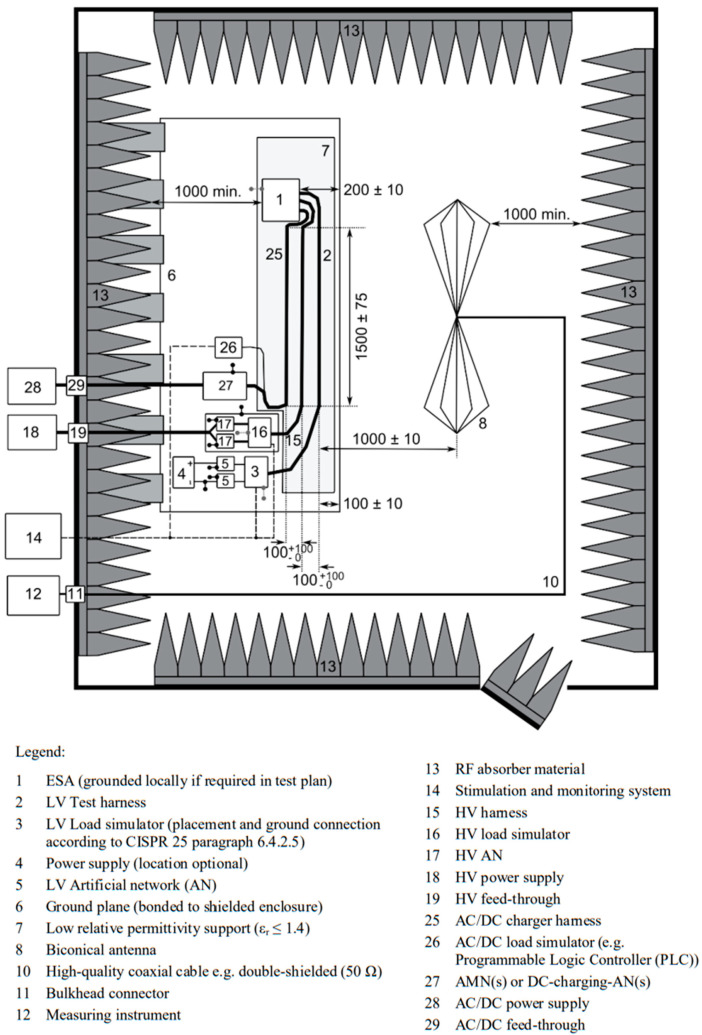
Diagram of the test site for measuring radiated electromagnetic emissions [5].

Conditions of the test object:

The test object must operate in normal mode, preferably under maximum load. Objects configured in the “grid-connected REESS charging mode” must be in charging mode during testing. When measuring across the entire frequency range, the charge state of the traction battery should be maintained between 20% and 80% of its maximum charge state. If the test is not performed with a REESS, the object shall be tested at its rated current. If the current consumption is adjustable, the current shall be set to at least 80% of its rated value. Tests were conducted at a test site that complied with the requirements specified in the normative documents. The test bench used for the measurements is shown in Figure 5, while the diagram of the test site is presented in Figure 6.

**Figure 7:**
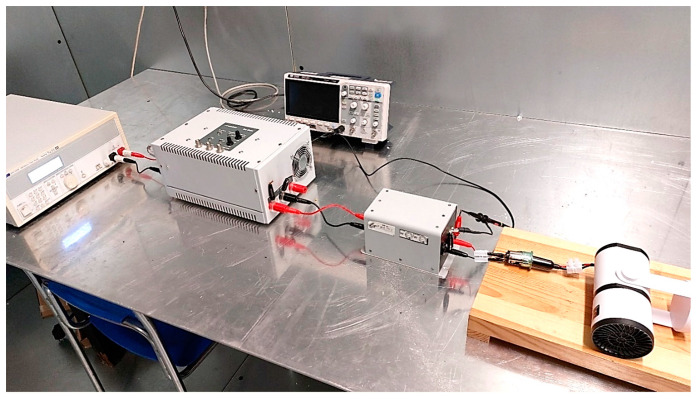
Photo of the test bench for measuring the emission of transient disturbances conducted along power cables (example of DUT: heating fan).

**Figure 8:**
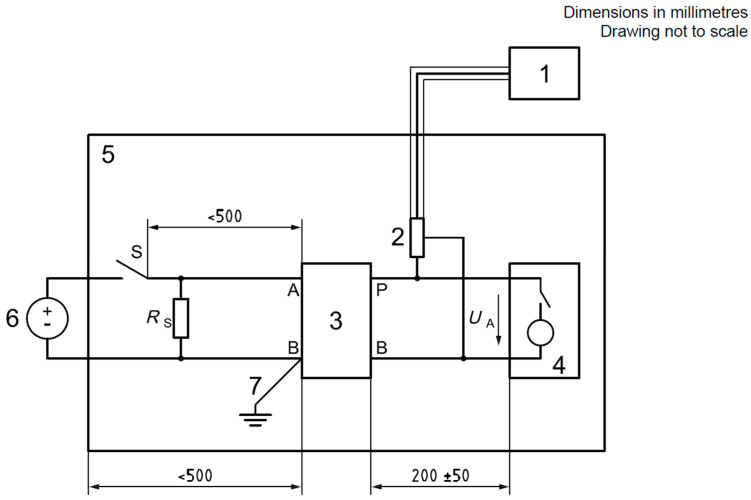
Diagram of the test setup for measuring the emission of transient disturbances conducted along power cables: 1 oscilloscope; 2 voltage probe; 3 artificial network; 4 PZE; 5 ground plane; 6 power source; 7 ground connection [5].

The parameters of the method for measuring transient emissions of conducted disturbances along power supply cables, in accordance with the requirements of UN Regulation No. 10 and ISO 7637-2 standard [26], are presented in Table 5 and Table 6. The tests were performed in compliance with the test setup specified in the normative documents. The test bench used for the measurements is shown in Figure 7, while the diagram of the test setup is presented in Figure 8. Measurement uncertainty was estimated at 1.40 dB.

#### 2.2.2. External Electromagnetic Environment in the Example of the City of Warsaw

Numerous studies discuss the measurement of electromagnetic fields in the environment [20,21,22,27,28], but none directly addresses the specific context of vehicles. The findings of such studies have not been analyzed in terms of their applicability for defining appropriate requirements for vehicles. Identifying the parameters of the operating environment is a crucial step in the subsequent verification of the correct operation of vehicles.

As part of the identification tests of the external electromagnetic environment, measurements were conducted in the city of Warsaw. The initial step in proper verification is identifying characteristic points where the highest electromagnetic field intensity values are expected. Based on our literature review, 16 characteristic points in the city were selected. The measurement points are listed in Table 7. Measurements were conducted using an RF EMF Strength Meter-EXTECH EMF Meter 480,846 (Teledyne FLIR, LLC 2, Paris, France). At each designated location, two values were recorded: peak and average values. The meter was positioned horizontally at a height of 1.5 m at each measurement point. The proposed measurement technique was successfully validated in a measurement scenario conducted in the EMC chamber and on the premises of the Łukasiewicz Research Network Automotive Industry Institute. The method was deemed reliable, with its uncertainty estimated at 1.77 V/m.

**Figure 9:**
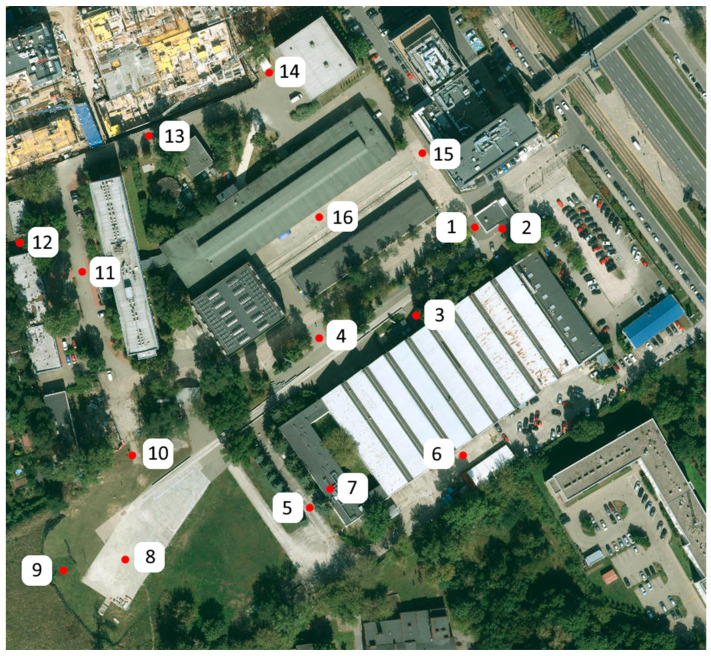
Measurement points on the premises of the Łukasiewicz Research Network Automotive Industry Institute.

Measurements were conducted using the same methodology in the vicinity of the Łukasiewicz Research Network Automotive Industry Institute to demonstrate differences in the intensity of the electromagnetic field within a smaller, verified area. The measurement points are presented in Figure 9.

## 3. Results

The results of the identification tests enable the characterization of the electromagnetic environment in modern vehicles operating on public roads. However, the absence of dedicated normative research methodologies in this field, combined with the reliance on methodologies derived from related areas, fails to provide a comprehensive answer to the question of vehicle safety in road traffic.

### 3.1. Results of Internal Electromagnetic Environment Measurements

**Figure 10:**
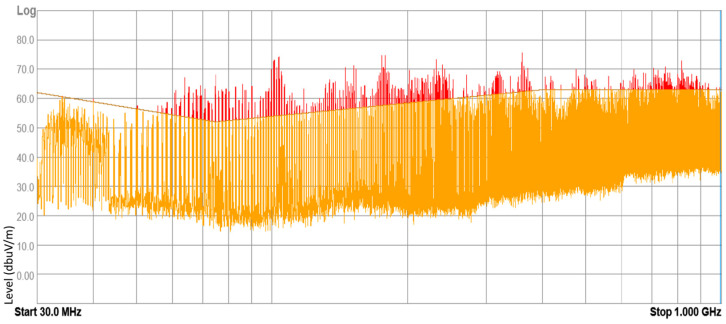
Result of radiated broadband electromagnetic interference emission—sample 1.

**Figure 11:**
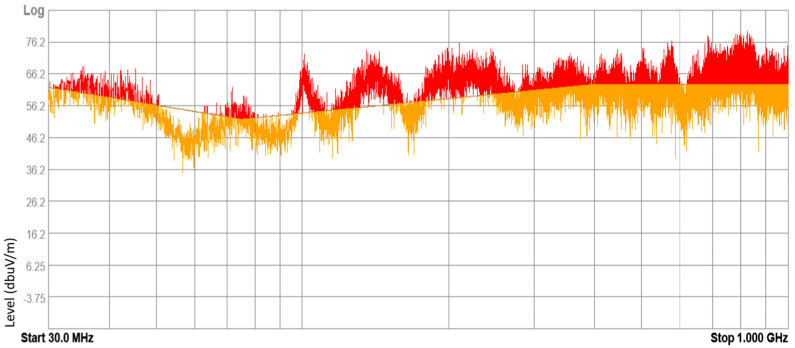
Result of radiated broadband electromagnetic interference emission—sample 2.

**Figure 12:**
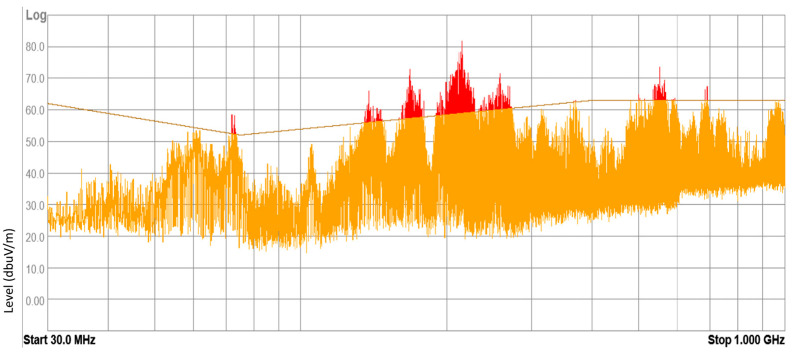
Result of radiated broadband electromagnetic interference emission—sample 3.

**Figure 13:**
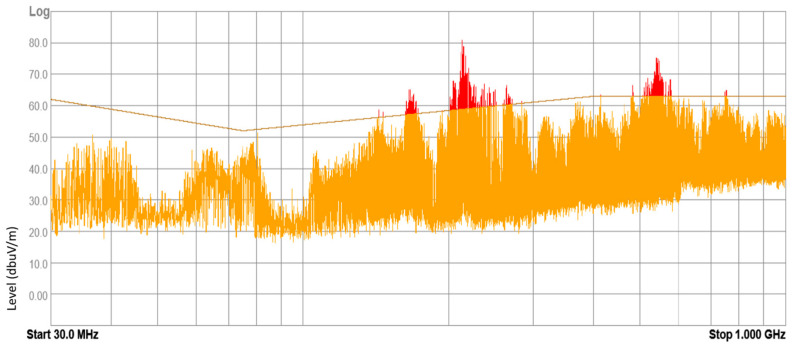
Result of radiated broadband electromagnetic interference emission—sample 4.

The devices under test (DUT) included electronic and electrical equipment available on the market for purchase and installed in vehicles currently in use. These devices included mobile chargers, FM transmitters, massage seat covers, heating fans, and DC-to-AC power converters. Tests were conducted on 43 samples of different DUTs, with approximately 20% of them yielding negative results according to the normative methodology. These results suggest that the electromagnetic environment inside a vehicle can pose a threat to other electronic equipment. Examples of radiated broadband electromagnetic interference emission results are presented in Figure 10, Figure 11, Figure 12 and Figure 13.

### 3.2. Results of External Electromagnetic Environment Measurements

Below are Table 8 and Table 9, which show the results obtained in Warsaw and at the Łukasiewicz Research Network Automotive Industry Institute, respectively.

## 4. Discussion

The conducted analysis presents the current internal and external electromagnetic environments of modern vehicles. Measurements were carried out under real-world conditions, reflecting the operational environment of autonomy systems currently being introduced to the market. The vehicle operating environment was analyzed with consideration of functional safety assumptions. The results indicate that, from the perspective of EMC considerations, the internal environment poses a greater threat than the external one, assuming a focus on unintentional threats. A separate issue, however, is deliberate electromagnetic attacks targeting vehicles and their electronic systems.

No new methods were introduced in the EMC measurements conducted during this research. Instead, normative recommendations were applied. However, the research purpose was innovative. Analysis revealed two key points: firstly, the study addressed a new application area (autonomous vehicles). Secondly, the scope of normative methods and requirements should be revised, particularly regarding measurement durations, frequency ranges, and levels of exposure to electromagnetic fields. This conclusion is based on the testing of vehicle components currently available on the market. Furthermore, obligatory tests should be expanded to encompass threats beyond EMC. It is also essential to highlight the influence of external conditions that degrade electrical systems, which may indirectly affect energy consumption, increase emissions, and reduce system immunity. A revised methodology should include verification tests after a specified period of operation, such as those performed during periodic vehicle inspections or technical tests.

## 5. Conclusions

The next step involves developing advanced electromagnetic compatibility (EMC) testing methodologies and criteria for assessing the functional safety and EMC of autonomy systems, including environment detection and analysis systems. Developing such methodologies is essential for the proper and safe integration of automated and autonomous vehicles into public road systems. Work has already commenced on a methodology that addresses the current shortcomings in EMC testing methods. The new methodology will encompass threats related to environmental factors such as temperature, vibrations, and humidity, as well as the degradation and wear of electronic components over time. The research process for developing this methodology includes conducting cyclical immunity tests on representative autonomy systems. The primary objective of this experimental research is to gain insights into potential functional losses in tested systems when exposed to various environmental and electromagnetic conditions.

Future works: Our primary focus lies in investigating the degradation of functionality and the alteration of electromagnetic compatibility (EMC) parameters in autonomous systems resulting from unintended cyclical exposures. Challenges remain in developing robust research methodologies, as well as in designing and implementing advanced testing facilities capable of simultaneously verifying multiple exposure scenarios. Furthermore, addressing intentional electromagnetic exposure, including electromagnetic attacks, and devising protective measures for autonomous vehicles against such threats, represent critical areas of ongoing research.

## Figures and Tables

**Table 1:** Scanning receiver parameters—broadband emission measurements.

Frequency Range	Peak Detector			Quasi-Peak Detector		
	Bandwidth	Step Size	Duration	Bandwidth	Step Size	Duration
	−6 dB			−6 dB		
30–1000 MHz	120 Hz	50 kHz	5 ms	120 Hz	50 kHz	1 ms

**Table 2:** Scanning receiver parameters—narrowband emission measurements.

Frequency Range	Average Detector		
	Bandwidth	Step Size	Duration
	−6 dB		
30–1000 MHz	120 Hz	50 kHz	5 ms

**Table 3:** Limit values—measurements of broadband emissions (requirements identical to those of the PN-EN 50498 standard [25]).

Granica E (dBμV/m) Przy Częstotliwości F (MHz)		
30–75 MHz	75–400 MHz	400–1000 MHz
E = 62 − 25.13 log (F/30)	E = 52 + 15.13 log (F/75)	E = 63

**Table 4:** Limit values—narrowband emission measurements (requirements identical to those of the PN-EN 50498 standard [25]).

Granica E (dBμV/m) Przy Częstotliwości F (MHz)		
30–75 MHz	75–400 MHz	400–1000 MHz
E = 52 − 25.13 log (F/30)	E = 42 + 15.13 log (F/75)	E = 53

**Table 5:** Supply voltages of objects.

	Nominal Voltage	Nominal Voltage
	12 V	24 V
Supply voltage	13.5 ± 0.5 V	27 ± 1 V

**Table 6:** Maximum allowed pulse amplitude (requirements identical to those of the PN-EN 50498 standard [25]).

Polarity of the Pulse Amplitude	Maximum Allowed Pulse Amplitude for	
	Vehicles with 12 V installation	Vehicles with 24 V installation
Positive	+75	+150
Negative	–100	–450

**Table 7:** List of measurement points in Warsaw.

Number	Adress
1	Parking P + R Żerań PKP, Marywilska
2	Transformer room, Gazownia, Warszawa
3	Intersection Maćka z Bogdańca z Nowo-Kowalskiego
4	Malborska 41
5	Nieświeska (E.ON Stacja Ładowania)
6	Zabraniecka 20
7	Oszmańska 20
8	Al. Jerzego Waszyngtona 2 B
9	Stanisława Rogalskiego
10	Church Śt. Jana z Dukli, Czerniakowska 2/4
11	Wilhelma Konrada Roentgena 5
12	Energetyczna 18
13	Mory 8
14	Estrady 50
15	Władysława Broniewskiego 44
16	Jagiellońska 79

**Table 8:** Measurement results in the city of Warsaw.

Measuring Point	MAX	AVR
	V/m	V/m
1	13.64	1.10
2	6.54	5.09
3	5.81	4.80
4	2.27	1.73
5	7.60	5.00
6	14.20	8.70
7	2.18	1.40
8	11.6	4.70
9	9.20	7.06
10	5.65	3.95
11	5.36	0.98
12	5.30	3.40
13	7.20	3.60
14	8.30	5.00
15	5.60	4.40
16	8.75	7.15

**Table 9:** Measurement results on the premises of the Łukasiewicz Research Network Automotive Industry Institute.

Measuring Point	MAX	AVR
	V/m	V/m
1	8.51	1.79
2	1.77	1.46
3	0.88	0.72
4	1.37	1.26
5	0.66	0.61
6	1.17	1.04
7	1.01	0.03
8	1.24	0.84
9	0.91	0.90
10	1.29	1.25
11	0.83	0.80
12	1.22	0.93
13	0.95	0.89
14	1.05	1.04
15	1.10	0.98
16	0.87	0.77

## Data Availability

Data are contained within the article.
